# A genome-wide association study reveals a locus for bilateral iridal hypopigmentation in Holstein Friesian cattle

**DOI:** 10.1186/s12863-017-0496-4

**Published:** 2017-03-29

**Authors:** Anne K. Hollmann, Martina Bleyer, Andrea Tipold, Jasmin N. Neßler, Wilhelm E. Wemheuer, Ekkehard Schütz, Bertram Brenig

**Affiliations:** 1 Institute of Veterinary Medicine, University of Goettingen, Burckhardtweg 2, 37077 Goettingen, Germany; 2 Pathology Unit, German Primate Center, Leibniz-Institute for Primate Research Goettingen, 37077 Goettingen, Germany; 3 Small Animal Clinic, University of Veterinary Medicine Hannover, 30559 Hannover, Germany

**Keywords:** Iris hypopigmentation, Cattle, Heterochromia iridis, Oculocutaneous hypopigmentation, Albinism, GWAS, Holstein Friesian

## Abstract

**Background:**

Eye pigmentation abnormalities in cattle are often related to albinism, Chediak-Higashi or Tietz like syndrome. However, mutations only affecting pigmentation of coat color and eye have also been described. Herein 18 Holstein Friesian cattle affected by bicolored and hypopigmented irises have been investigated.

**Results:**

Affected animals did not reveal any ophthalmological or neurological abnormalities besides the specific iris color differences. Coat color of affected cattle did not differ from controls. Histological examination revealed a reduction of melanin pigment in the iridal anterior border layer and stroma in cases as cause of iris hypopigmentation. To analyze the genetics of the iris pigmentation differences, a genome-wide association study was performed using Illumina BovineSNP50 BeadChip genotypes of the 18 cases and 172 randomly chosen control animals. A significant association on bovine chromosome 8 (BTA8) was identified at position 60,990,733 with a -log_10_(*p*) = 9.17. Analysis of genotypic and allelic dependences between cases of iridal hypopigmentation and an additional set of 316 randomly selected Holstein Friesian cattle controls showed that allele A at position 60,990,733 on BTA8 (*P* = 4.0e–08, odds ratio = 6.3, 95% confidence interval 3.02–13.17) significantly increased the chance of iridal hypopigmentation.

**Conclusions:**

The clinical appearance of the iridal hypopigmentation differed from previously reported cases of pigmentation abnormalities in syndromes like Chediak-Higashi or Tietz and seems to be mainly of cosmetic character. Iridal hypopigmentation is caused by a reduced content of melanin pigment in the anterior border layer and iridal stroma. A single genomic position on BTA8 was detected to be significantly associated with iridal hypopigmentation in examined cattle. To our knowledge this is the first report about this phenotype in Holstein Friesian cattle.

**Electronic supplementary material:**

The online version of this article (doi:10.1186/s12863-017-0496-4) contains supplementary material, which is available to authorized users.

## Background

For many decades, eye color and eye color genetics has been an intensively studied field of research in humans and other species. More than 100 years ago, a relatively plain dominant-recessive model of inheritance of human brown and blue eye color has been assumed [1]. However, in recent years it became clear that iris pigmentation is under the control of a plethora of genes and is rather a quantitative than a simple Mendelian trait [2, 3].

Pigmentation of the eye, skin, and hair is the result of melanin pigment production in melanocytes. Melanin producing cells contain specialized lysosome-related organelles, the melanosomes, depositing melanin pigment in mature stage. Brighter colored eyes usually have less melanin pigment in iris stroma than darker eyes [2, 4, 5]. This and other factors as pigmentation of the posterior pigment epithelium [5], the type of melanin (eu- or pheomelanin) in iris melanocytes [5], light-scattering, and absorption processes [4] are believed to influence human eye color determination.

Changes in iris color have also been reported in cattle. Discolorations of the iris, either mono- or bilateral, complete or partial, are usually referred to as heterochromia iridis (HI). However, the phenotypic appearance differs remarkably between reported cases. The most prominent iris color variations were detected in cattle suffering from complete albinism, showing a pale blue iris with a white periphery [6–8]. Less distinct pigmentation anomalies were observed in non-albinotic HI cases, showing a bicolored iris with a central ring of blue and a peripheral ring of gray or brown [9, 10]. Besides albinism, severe eye color changes in cattle were also observed in syndromes like Tietz [11] and Chediak-Higashi [12, 13]. These syndrome related pigmentation alterations are usually accompanied by more restrictive anomalies. German Fleckvieh cattle with Tietz like syndrome exhibited bilateral deafness and colobomatous eyes [11]. Chediak-Higashi syndrome usually manifested in bruisability and bleeding tendency [12, 13]. In albino cases with HI further clinical features related to eye development like nystagmus and blindness were detected [8].

Recently an alteration of iris coloration has been observed in Angus and Simmental breed. Affected cattle showed an oculocutaneous hypopigmentation (OH) with a pale blue iris and a tan periphery coupled with a change in coat color from black to chocolate. It is assumed that this aberration was introduced into the Simmental breed in the late 1950s by Angus founders and is inherited as an autosomal recessive trait. An amino acid exchange in the Ras-related Protein Rab-38 (*RAB38*) gene was identified as the disease causing mutation (Jon Beever, personal communication). Likewise, *Rab38* ^*cht*^ */Rab38* ^*cht*^ mice with a mutation (G146T) in exon 1 of *Rab38* develop a similar phenotype with chocolate coat color and ocular hypopigmentation [14, 15].

In the current study the clinical, histological and molecular examination of bilateral iris hypopigmentation in 18 Holstein Friesian cattle are described. The aim of the study was the description of the phenotype, the identification of pigmentation alterations in the eye of affected cattle and the determination of the underlying genetics of iridal hypopigmentation in cattle.

## Methods

### Animals, pedigree information and DNA samples

A total of 18 Holstein Friesian (HF) cattle (9 male, 9 female) with hypopigmented irises originating from eight different farms were used for this study. Pedigree data were obtained from the German livestock database service provider (VIT) and checked for shared common ancestors. Complete pedigree data were available for ten individuals, while for eight animals only paternal pedigree data were present. Pedigrees of HF cases were constructed using Pedigraph [16].

DNA was extracted from EDTA-blood using MagNa Pure LC DNA Isolation Kit I (Roche Diagnostics Deutschland GmbH, Mannheim, Germany). Control DNA was obtained from the depository at the Institute of Veterinary Medicine (Göttingen, Germany).

### Histology

To determine the exact cause of the hypopigmentation, histological evaluation of irises of unaffected and affected animals was conducted. Eyes of each animal were completely enucleated immediately after slaughtering. The dorsal half of each freshly enucleated eyeball was opened by incision of the sclera midway between the cornea and the optic nerve. After removal of the vitreous body, eyeballs were immersed in 4% phosphate-buffered formaldehyde. After fixation for at least 48 h, lenses were removed and four cross sections were made through the anterior half of the eyeballs including dorsal, medial, ventral, and lateral aspects of the iris and adjacent structures of the anterior eye including ciliary body and cornea. Additionally, a cross section was prepared from the lens and from the caudal half of the eyeball at the level of the optic nerve. Trimmed tissue samples were paraffin-embedded, sectioned at 3 μm, and stained with hematoxylin and eosin (HE) for light microscopic examination.

### Genotyping, genome-wide association study (GWAS) and statistical analysis

For GWAS 172 randomly selected HF cattle genotypes were used for GWAS. The 18 cases of iridal hypopigmentation were also genotyped using the Illumina BovineSNP50 BeadChip. Final reports were generated using GenomeStudio V2011.1 (Illumina, San Diego, USA) and imported into SNP & Variation Suite (SVS) 8.5.0 (Golden Helix, Bozeman, USA). Genotype data were screened through a series of quality control criteria, including Mendelian errors, minor allele frequency (MAF) < 1%, *p*-value of Fisher’s Hardy-Weinberg equilibrium (HWE) test < 0.001 (based on controls), and single nucleotide polymorphism (SNP) call rate < 98% reducing the data set from 54,610 to 44,952 SNPs. Associations were calculated under an additive, recessive, and dominant model [17]. The additive genetic model fitted the data best. We did not detect significant evidance for population stratification. Genomic positions refer to NCBI UMD3.1.1.

Genotypic and allelic dependences were calculated between the 18 cases of iridal hypopigmentation and an additional set of randomly chosen 316 controls. Genotypes of this validation cohort were extracted from another data set generated using the Illumina BovineSNP50 BeadChip. Genotypes were compared using 3×2 or 2×2 contingency tables and Fisher’s exact or *χ* ^2^ statistics (df = 2). *P* < 0.005 was considered to be significant. Calculations were done using Microsoft Excel for Mac 2011 (14.7.1). HWE *χ* ^2^-values were calculated according to Rodriguez et al. [18] and considered significant with *p* > 0.05 (df = 2).

A haplotype association test was performed using a moving window of five markers. The expectation-maximization algorithm was applied using 50 iterations and a convergence tolerance of 0.0001 [19]. Results were corrected for multiple testing according to Bonferroni.

### Polymerase chain reaction (PCR) and sanger sequencing of *RAB38*

*RAB38* primers were designed using the online software tool Primer-Blast [20] with the following sequences RAB38_Ex1_F: 5’-CTTCCCGGGTCCGCAG-3’, RAB38_Ex1_R: 5’-CTGGCACAGGAGATGGTCTG-3’, RAB38_Ex2_F: 5’-ACTTTGCGGAGTGATCTGCT-3’, RAB38_Ex2_R: 5’-GCTGCCTTAGCCACAAACAC-3’, RAB38_Ex3_F: 5’-CATGGGACAGGGTTTAGAAAGAGA-3’, RAB38_Ex3_R: 5’-AGGCATAGGTCTTTGGCTTG-3’. Primers were designed to amplify the complete coding region including splice sites of *RAB38*. All primers were synthesized by Sigma-Aldrich (Steinheim, Germany). PCR was performed in a total volume of 25 μl using FastStart *Taq* DNA Polymerase, dNTPack (Roche Diagnostics, Mannheim, Germany). One reaction mix (25 μl) included 1.5 U Faststart *Taq* DNA Polymerase, 200 μmol/L dNTP, 0.4 μmol/L of each primer, 1× PCR reaction buffer (including 20 mM MgCl_2_), 1× Q-Solution (Qiagen, Hilden, Germany) and 40 ng of DNA. Cycling conditions were 95 °C for 10 min, followed by 30 cycles of 95 °C for 30 s, 60 °C for 30 s and 72 °C for 30 s. Final elongation step was 72 °C for 5 min. PCR products were sequenced with the BigDye Terminator v3.1 Cycle Sequencing Kit (Applied Biosystems, Fisher Scientific GmbH, Schwerte, Germany) on an ABI PRISM 3130xl Genetic Analyzer (Life Technologies, Foster City, USA) after purification using Rapid PCR Cleanup Enzyme Set (New England Biolabs GmbH, Frankfurt am Main, Germany). As reference sequence of *RAB38* assembly UMD3.1.1 with accession number AC_000186.1 was used.

## Results

### Comprehensive clinical examination of affected cattle

**Fig. 1:**
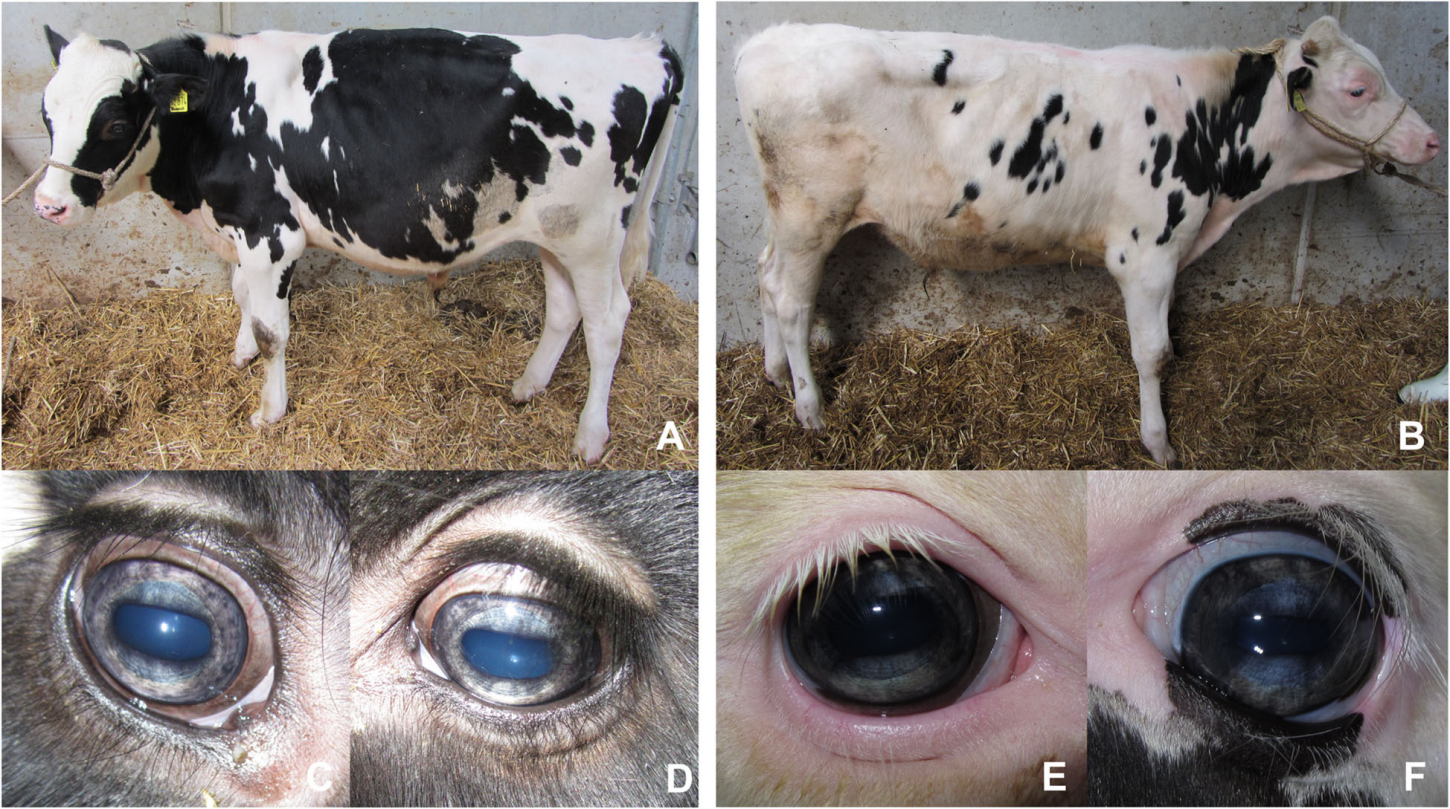
Phenotypic appearance of iridal hypopigmentation. HF cattle were ophthalmological and neurological examined and irises underwent histologic evaluation. **a** and **b**: *Coat color* of affected cattle was typical for the breed and without any sign for albinism. Both cattle were normally developed at time of examination. **c**-**f**: *Iris color* of cases **a** and **b**. The degree of discoloration clearly differed between cases. All affected cattle showed a *bicolored iris* with a central ring of *silver-blue* and a peripheral ring of *brown-gray. Iris color* within one iris showed alternating *darker* and *lighter parts*

As shown in Fig. 1, affected HF cattle had a normal, breed specific coat color with no obvious color deviations of eyelids and eyelashes. Furthermore, cases did not show any neurological deficits, i.e. disturbance of the level of consciousness, mentation and behaviour, posture and gait, postural reactions, as well as spinal and cranial nerve function [21]. Signs of a Horner syndrome, which has been described in conjunction with HI in humans, were absent [22, 23]. Pupillary light and blink reflex were normal. No spontaneous nystagmus or strabismus could be observed. The animals had a physiological nystagmus. The ocular fundus was normal, a retinitis pigmentosa was excluded. To exclude common infectious diseases, animals were also tested for bovine viral diarrhea virus (bovine virus diarrhea), bovine herpesvirus type 1 virus (infectious bovine necrotic rhinotracheitis), bovine leukaemia virus (bovine lymphomatosis), *Brucella* spp. (bovine brucellosis), bluetongue virus (bluetongue disease), *Mycobacterium paratuberculosis* (paratuberculosis), Schmallenberg virus and *Neospora caninum* (bovine neosporosis). All test results were negative. Summarizing the clinical analysis of the examined animals, a bilateral hypopigmentation caused by a hitherto unknown genetic variation strictly affecting iridal coloration was suspected. As the affected animals did not show any other anomalies, syndromes like Chediak-Higashi or Tietz were excluded.

Detailed ophthalmological examination of the irises of affected animals showed two merging, but clearly differentiable shades of color, a bluish center and a grayish peripheral ring. Although all animals showed a clear discoloration of the iris, there was considerable variation of iris coloration between animals. The color of the central iridal parts ranged from silvery-blue to gray-blue with darker and lighter parts. In the periphery, irises were light brown to gray with occasional light gray zones (Fig. 1). In some animals, the degree of discoloration differed within the peripheral iris and showed alternating darker and lighter regions. Partial brownish corneoscleral pigmentation was visible in a few animals.

### Determination of common ancestors

To identify a potential founder of the eye color phenotype, a pedigree analysis was performed. Available pedigree data of HF cattle revealed that the 18 cases descended from 11 different sires whereas dams were not closely related. None of the dams had been reported to show the typical iris hypopigmentation. In total 10 male ancestors, born between 1954 and 1983, were identified being present in the dam and sire line of every case. The available pedigree data did not reveal a single common founder for HF cases, and HF cases were not closely related.

### Histological examinations

**Fig. 2:**
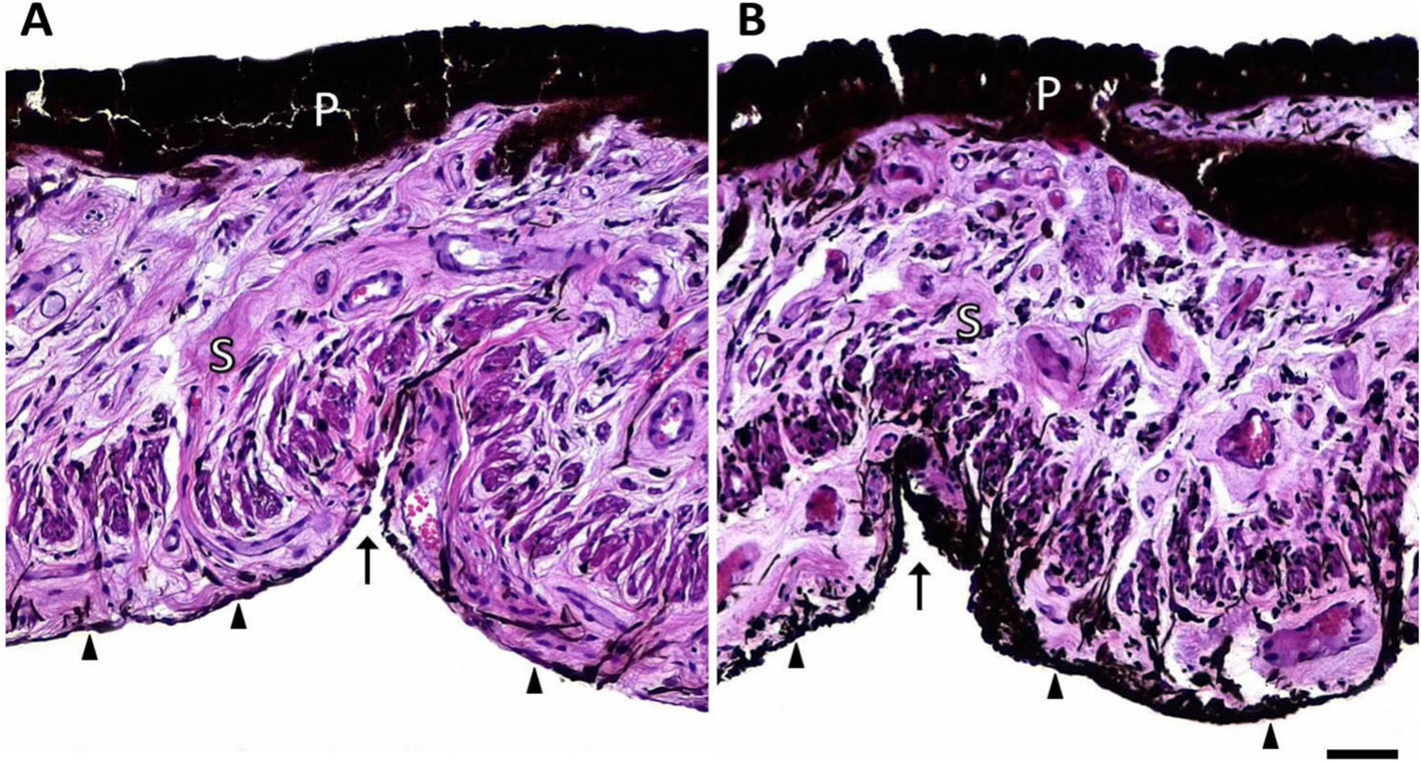
Histological cross section through the ciliary zone of the left ventral iris near the collarette with characteristic Fuchs’ crypts. Comparison of the pigmentation of the three different iris layers anterior border layer (*arrowheads*), stroma (*S*), and posterior pigmented epithelium (*P*) of affected (**a**) and control animals (**b**). A distinct hypopigmentation of the anterior border layer (*arrowheads*) and the iridal stroma (*S*) in the affected animal (**a**) can be observed compared to the unaffected control animal (**b**). Thickness and pigment content of the double-layered posterior pigmented epithelium (*P*) is similar in both animals. HE staining, *scale bar* = 50 μm

Histological evaluation of irides revealed less melanin pigment deposition in the anterior border layer and the iridal stroma in the affected animals compared to the normal iris of the unaffected control animals (Fig. 2). This form of hypopigmentation was evident in all examined localizations of the irises in the affected animals. Only the degree of hypopigmentation varied between the different analysed regions and within irises at the same locations. In general, hypopigmentation seemed to be more pronounced in central iridal parts (pupillary zone and central parts of the ciliary zone) and in the dorsal as well as ventral iris. Iridal thickness, stromal density, and cellular composition were consistent in all examined animals. Differences in pigmentation of the posterior pigmented epithelium of the iris, other uveal structures (ciliary body, choroid), as well as of the retinal pigment epithelium were not detected. All other examined ocular structures were inconspicuous.

### Determination of associated chromosomal regions

**Fig. 3:**
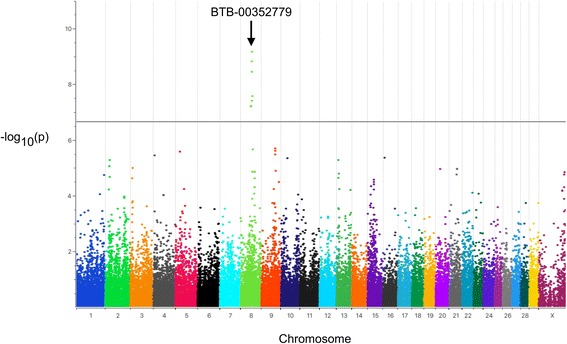
Manhattan plot of -log_10_(*p*)-values for SNPs by genomic location (NCBI UMD3.1.1). Calculations were performed using SNP data of 18 cases and 172 randomly selected control HF cattle. The *black line* indicates the Bonferroni genome-wide significance level of -log_10_(*p*) = 6.65 at *p* < 0.01. The marker with the highest -log_10_(*p*)-value is indicated (BTB-00352779)

To identify associated chromosomal regions a genome-wide association study was performed. The 18 cases were compared to 172 unrelated control cattle. Seven highly associated SNPs above the Bonferroni genome-wide significance level were identified on bovine chromosome 8 (BTA8) spanning from 57.3 to 65.3 Mb (Fig. 3; Additional file 1: Table S1). The SNP with the highest -log_10_(*p*) = 9.17 (BTB-00352779) was located at position 60,990,733 (NCBI UMD3.1.1). It is noteworthy that six genes, i.e. *CLTA*, *GNE*, *RNF38*, *SHB*, *TRIM14*, and *NANS*, located in the region from 57.3 to 65.5 Mb on BTA8 have been associated with earlobe color in chicken [24]. However, so far none of these genes have been reported to be directly involved in eye pigmentation.

To validate the identified associations and to determine the genotypic and allelic dependences compared with an unrelated additional control cohort, 316 Holstein Friesian cattle were randomly selected from a previously generated data set and compared with the genotypes of the 18 cases. Table 1 summarizes the results and statistics of the comparison of the genotypes. Both groups (cases/controls) were in Hardy-Weinberg equilibrium at BTB-00352779 (cases: *χ* ^2^ = 5.8, controls: *χ* ^2^ = 0.72). However, as determined by using a 3x2 contingency table and Fisher’s exact statistics genotypes were significantly not independent at BTB-00352779 with *P* = 6.19e-07. To evaluate which allele was associated with a higher risk of developing an iridal hypopigmentation, odds ratios were calculated (Table 2). As shown in Table 2 alleles were significantly dependent using a 2x2 contingency table and *χ* ^2^-statistics. The presence of the A-allele was leading to a 6.3-times higher chance to develop an iridal hypopigmentation (95% CI: 3.0172–13.1727). Using a moving window of five SNPs a haplotype association was calculated for the 18 cases. Highly significant associations were detected for five haplotypes including BTB-00352779 and flanking markers (Table 3). Haplotype AAAAA with BTB-00352779 as first marker showed the highest odds ratio (OR = 8.31; 95% CI: 3.62–19.08).

**Table 1:** Genotypic dependences of iridal hypopigmentation

SNP (chromosome)	Genotype	Cases (*n* = 18)			Controls (*n* = 316)			*P*-value
		Obs.	Exp.	HWE	Obs.	Exp.	HWE	
BTB-00352779 (BTA8)	CC	5	14.5		265	254.9		
	AC	13	3.4		50	60.2		
	AA	0	0.1	5.8	1	0.9	0.72	6.19e-07

*Abbreviations*: *HWE* Hardy-Weinberg equilibrium *χ* ^2^-value (*p* > 0.05, df = 2), *Obs.* observed number of genotypes, *Exp.* expected number of genotypes, *P-value* determined using a 3 × 2 contingency table and Fisher’s Exact statistics (two-tailed)

**Table 2:** Allelic dependences of iridal hypopigmentation

SNP (chromosome)	Allele	Cases (*n* = 18)		Controls (*n* = 316)		*χ* ^2^	*P*-value	OR	0.95 CI
		Obs.	Exp.	Obs.	Exp.				
BTB-00352779 (BTA8)	C	23	32.5	580	570.5			0.16	0.076–0.3314
	A	13	3.5	52	62.5	30.15	4.0e–08	6.3	3.0172–13.1727

*Abbreviations*: *Obs.* Observed number of alleles, *Exp.* expected number of alleles, *χ* ^*2*^ *p-value χ* ^2^- and *p*-values were determined using a 2 × 2 contingency table and *χ* ^2^-statistics, *OR* odds ratio, *0.95 CI* 95% confidence intervals

**Table 3:** Haplotype association analysis for markers flanking BTB-00352779

First Marker ^a^	Position ^b^	Haplotype	-log_10_(*p*)	(*p*)_B_^c^	OR ^d^	0.95 CI ^e^
ARS-BFGL-NGS-40630	60800770	AGAGC	0.57	1	0.65	0.30–1.40
		GAGGC	0.02	1	1.02	0.46–2.27
		GGAGC	1.16	1	0.28	0.07–1.20
		AAAGA	7.63	0.0085	7.90	3.46–18.04
		AGAAC	0.15	1	0.67	0.09–5.24
		AAAGC	0.54	1		
		AAGGC	0.45	1	0.04	
ARS-BFGL-NGS-115798	60823250	GAGCA	0.52	1	0.67	0.31–1.44
		AGGCA	0.10	1	0.90	0.40–2.00
		GAGCC	1.21	1	0.27	0.06–1.17
		AAGAA	7.62	0.0087	7.90	3.46–18.04
		GAACA	0.19	1	0.61	0.08–4.78
		AAGCC	0.54	1		
ARS-BFGL-NGS-12436	60872538	AGCAA	0.51	1	0.67	0.31–1.45
		GGCAA	0.10	1	0.90	0.40–1.99
		AGCCG	1.57	1	0.22	0.05–0.95
		AGAAA	7.62	0.0087	7.90	3.46–18.04
		AACAG	0.19	1	0.61	0.08–4.78
ARS-BFGL-NGS-102	60952836	GCAAA	0.68	1	0.64	0.31–1.30
		GCCGA	0.71	1	0.39	0.09–1.70
		GAAAA	7.72	0.0069	7.99	3.50–18.23
		GCCGG	1.14	1		
		ACAGA	0.19	1	0.62	0.08–4.83
BTB-00352779	60990733	CAAAG	0.76	1	0.61	0.30–1.25
		CCGAG	0.68	1	0.40	0.09–1.75
		AAAAA	7.97	0.0038	8.31	3.62–19.08
		CCGGG	1.12	1		
		CAGAG	0.18	1	0.64	0.08–4.96

^a^ A moving window of 5 SNPs was used to determine haplotype associations. Each haplotype begins with the listed first SNP followed by four of the following consecutive SNPs: ARS-BFGL-NGS-40630, ARS-BFGL-NGS-115798, ARS-BFGL-NGS-12436, ARS-BFGL-NGS-102, BTB-00352779, Hapmap39688-BTA-81545, Hapmap49326-BTA-81546, ARS-BFGL-NGS-55438, Hapmap36177-SCAFFOLD210634_2319

^b^ Positions according to NCBI UMD3.1.1

^c^ Bonferroni corrected *p*-value

^d^ OR: Odds ratio

^e^ 95% confidence interval upper and lower limit

### Exclusion of *RAB38* as candidate for bilateral iridal hypopigmention

Although the analysed cases of iridal hypopigmentation did not show the typical phenotype described for oculocutaneous hypopigmentation (OH) in Angus and Simmental cattle, i.e. eyes with pale blue irises around the pupil, a tan periphery and slightly bleached black coats looking grey or red, bovine *RAB38* was screened for mutations in the 20 cases and controls. In addition, *RAB38* is located on BTA29 which did not show any associated SNP in the GWAS. However, to exclude *RAB38* as causative for iridal hypopigmentation coding regions including splice sites were amplified and fragments sequenced. As expected no disease causing polymorphisms or alterations from the reference genome for cases and controls were detected.

## Discussion

Iridal hypopigmentation in cattle is attributed to a reduced content of melanin pigment in the anterior border layer and iridal stroma. These findings clearly differ from previously reported histological observations in HI cases, showing a reduction in eye pigmentation in different uveal structures as different iris layers, retinal pigment epithelium (RPE) [8], and choroid [6, 25]. Leipold and Huston observed a non-albinotic case of HI with reduced iris pigmentation in anterior border layer, iris stroma, and posterior pigment epithelium in Hereford cattle. Pigmentation in the remaining pigmented eye structures was also reduced and iridal stroma was hypoplastic [25]. Iris hypopigmentation in cases was exclusively located in the anterior border layer and the iridal stroma, and no structures were fully devoid of pigment. Pigmentation differences as seen in the present cases of iris hypopigmentation seemed to be rather comparable with naturally occuring eye color variances in humans than to HI. Human eye color variance is mainly due to differences in the amount of melanin pigment in the anterior border layer and iridal stroma. Pale colored eyes generally contain less melanin compared to brown eyes [2, 5].

In cattle with iris hypopigmentation, gradual iridal brightening can be explained by a reduced melanin content of variable intensity affecting the anterior border layer and the iridal stroma, resulting in blue to gray-brown instead of the normal black color. Differences in coloration between central and peripheral parts of the iris, namely the rather bluish color of the central iridal ring and the gray to light brown color of peripheral iridal parts, are probably attributed to the increasing thickness of the iris towards the periphery, associated with increased amounts of collagen in the peripheral iridal stroma. As the amount of collagen fibres within the iridal stroma is known to be another important determining factor of eye color, at least in humans [2], it may be assumed that variations in iris brightening between central and peripheral iridal parts in cattle with iris hypopigmentation result from differences in the amount of collagen and, thus, in the backscattering properties of the respective iridal regions. In this respect it is noteworthy that in the associated region on BTA8 collagen gene *COL15A1* is located at position 64,437,276–64,540,739 which has been shown to be expressed in multiple ocular structures [26]. In contrast, the amount of collagen in the iridal stroma of normal dark brown to black-eyed cattle seems to have a minor influence on iris coloration as almost all light is probably absorbed by the extensive eumelanin deposits in the anterior border layer and the iridal stroma accounting for a homogenous dark eye color [27, 28]. Variations of the degree of discoloration within the peripheral iris with alternating darker and lighter regions in affected animals of the present study are caused by regional differences in the melanin content of the anterior border layer and the iridal stroma, which were confirmed by histological analyses.

Taking all clinical findings together, there were no other abnormalities found in conjunction with the iris hypopigmentation. Although no long-term effects were examined, iris hypopigmentation seems to be mainly of cosmetic character. Under physiological conditions melanin pigment protects from ultraviolet (UV) light, and humans with lighter eye color seem to be more susceptible to age related macula degeneration [29] and uveal melanoma [30, 31]. This is comparable with blue-eyed horses that are at higher risk to develop ocular squamous cell carcinoma [32].

## Conclusion

The bilateral iridal hypopigmentation in HF cattle described here was due to a reduction of melanin pigment in the anterior border layer and iridal stroma. The phenotype was highly associated with a chromosomal region on BTA8. Haplotype association analysis showed that the presence of the A-alleles at the associated SNPs significantly increased the chance of developing an iridal hypopigmentation.

## Additional file

Additional file 1: Table S1. Details of markers on BTA8 associated with iridal hypopigmentation in HF cattle. (DOCX 44 kb)

## Abbreviations

BTA: Bos taurus chromosome
CI: Confidence interval
df: Degrees of freedom
GWAS: Genome-wide association study
HF: Holstein Friesian cattle
HI: Heterchromiasis iridis
MAF: Minor allele frequency
OH: Oculocutaneous hypopigmentation
OR: Odds ratio
SNP: Single nucleotide polymorphism
