# Supplementary material for: A genome-wide association study reveals a locus for bilateral iridal hypopigmentation in Holstein Friesian cattle
Source: BMC Genet. 2017 Mar 29;18:30. doi: 10.1186/s12863-017-0496-4 (PMC5372310; doi:10.1186/s12863-017-0496-4)
Supplement: Additional file 1: Table S1. — Details of markers on BTA8 associated with iridal hypopigmentation in HF cattle. (DOCX 44 kb) [file 12863_2017_496_MOESM1_ESM.docx]

| Marker | Position^1)^ | Obs. -log_10_(p)^2)^ | Exp. -log_10_(p) |
| --- | --- | --- | --- |
| BTA-108808-no-rs | 57307140 | 7.21 | 3.91 |
| BTB-00349451 | 57403486 | 7.2 | 3.84 |
| BTB-00352779 | 60990733 | 9.17 | 4.95 |
| Hapmap36177-SCAFFOLD210634_2319 | 61115517 | 8.81 | 4.47 |
| Hapmap40026-BTA-81432 | 61213362 | 8.44 | 4.25 |
| BTB-00351461 | 61777605 | 7.39 | 3.99 |
| BTA-19347-no-rs | 65295376 | 7.54 | 4.10 |

1) Positions according to NCBI UMD3.1.1; 3) Bonferroni genome-wide significance level of -log_10_(p) = 6.65 (p < 0.01)
